# Atherosclerosis from Newborn to Adult—Epidemiology, Pathological Aspects, and Risk Factors

**DOI:** 10.3390/life13102056

**Published:** 2023-10-14

**Authors:** Alina Costina Luca, Simona Georgiana David, Alexandru Gabriel David, Viorel Țarcă, Ioana-Alexandra Pădureț, Dana Elena Mîndru, Solange Tamara Roșu, Eduard Vasile Roșu, Heidrun Adumitrăchioaiei, Jana Bernic, Elena Cojocaru, Elena Țarcă

**Affiliations:** 1 Pediatrics Department, “Grigore T. Popa” University of Medicine and Pharmacy, 700115 Iasi, Romania; alina.luca@umfiasi.ro (A.C.L.); mindru.dana@umfiasi.ro (D.E.M.); eduard.rosu@umfiasi.ro (E.V.R.); 2 Saint Mary Emergency Hospital for Children, 700309 Iasi, Romania; simona.david23@gmail.com (S.G.D.); alexandru.david@sfmaria-iasi.ro (A.G.D.); paduret.alexandra@gmail.com (I.-A.P.); ad.heidi91@gmail.com (H.A.); 3 Department of Preventive Medicine and Interdisciplinarity, “Grigore T. Popa” University of Medicine and Pharmacy, 700115 Iasi, Romania; 4 Nursing Department, “Grigore T. Popa” University of Medicine and Pharmacy, 700115 Iasi, Romania; solange.rosu@umfiasi.ro; 5 Discipline of Pediatric Surgery, “Nicolae Testemițanu” State University of Medicine and Pharmacy, 2025 Chisinau, Moldova; jana.bernic@usmf.md; 6 Department of Morphofunctional Sciences I—Pathology, “Grigore T. Popa” University of Medicine and Pharmacy, 700115 Iasi, Romania; 7 Surgery II Department—Pediatric Surgery, “Grigore T. Popa” University of Medicine and Pharmacy, 700115 Iasi, Romania; tarca.elena@umfiasi.ro

**Keywords:** atherosclerosis, epidemiology, pathophysiology, dyslipidemia, childhood

## Abstract

Cardiovascular disease is the leading cause of mortality and morbidity throughout the world, accounting for 16.7 million deaths each year. The underlying pathological process for the majority of cardiovascular diseases is atherosclerosis, a slowly progressing, multifocal, chronic, immune-inflammatory disease that involves the intima of large and medium-sized arteries. The process of atherosclerosis begins in childhood as fatty streaks—an accumulation of lipids, inflammatory cells, and smooth muscle cells in the arterial wall. Over time, a more complex lesion develops into an atheroma and characteristic fibrous plaques. Atherosclerosis alone is rarely fatal; it is the further changes that render fibrous plaques vulnerable to rupture; plaque rupture represents the most common cause of coronary thrombosis. The prevalence of atherosclerosis is increasing worldwide and more than 50% of people with circulatory disease die of it, mostly in modern societies. Epidemiological studies have revealed several environmental and genetic risk factors that are associated with the early formation of a pathogenic foundation for atherosclerosis, such as dyslipidemia, hypertension, diabetes mellitus, obesity, and smoking. The purpose of this review is to bring together the current information concerning the origin and progression of atherosclerosis in childhood as well as the identification of known risk factors for atherosclerotic cardiovascular disease in children.

## 1. Introduction

Cardiovascular disease (CVD) is a public health problem, being a major cause of mortality and morbidity throughout the world [1,2,3]. The underlying cause of the majority of cardiovascular disease is atherosclerosis, a complex disease whose etiology is multifactorial and which typically is a slowly progressing chronic disorder of medium-sized and large arteries that manifests clinically through thrombotic events [4,5]. For many years, it was believed that atherosclerosis consisted mainly of a passive process of accumulation of low-density lipoprotein (LDL) cholesterol, apolipoprotein B, and lipoprotein (a) in the vessel wall [6,7]. In the past decades, it became apparent that atherosclerosis is, in fact, a chronic inflammatory disease, and the risk for its clinical sequelae is associated with the elevation of inflammation markers (measured by levels of C-reactive protein or myeloperoxidase activity) and high LDL [8]. Moreover, recent studies suggest that both adaptive and innate immune mechanisms can modulate the progression of the disease. Among the autoantigens identified in atherosclerotic lesions, oxidized LDL (oxLDL) has a prominent role [9,10]. 

Although atherosclerosis manifests clinically in middle and late adulthood, it is well known that it has a long asymptomatic phase of development, which starts in childhood as an accumulation of foam cells and T lymphocytes in the intima of the arteries. In most children, atherosclerotic vascular changes are minor and can be minimized or prevented with a healthy lifestyle. The incidence of childhood obesity has increased two-fold. Half of all overweight and obese children developed adverse cardiovascular risk factors at a mean age of 12.6 years, exponentially increasing their risks of cardiovascular disease in later life [11,12]. 

## 2. Epidemiology

Cardiovascular disease is the leading cause of disability and premature mortality worldwide, which is why it is called “the disease of the century” with more than 50% of people dying of circulatory pathology [13]. According to epidemiological studies up to 2020, CVD affected 422.7 million people and caused over 17 million deaths worldwide in 2015, representing 31% of all global deaths. Globally, over 75% of CVD-related deaths occur in low- and middle-income countries because of the limited access to healthcare services, which can delay the detection of the atherosclerosis process until the symptomatic phase, thus increasing premature mortality from CVD [1,14]. In high-income countries, the prevalence and incidence of CVD have increased as a result of tobacco use, high alcohol use, physical inactivity, and unhealthy diet [15].

Atherosclerosis, the main pathological process of most cardiovascular diseases, is predominantly an asymptomatic condition, and it is difficult to determine the incidence accurately. In addition, atherosclerosis can begin in childhood and remain latent and asymptomatic for many years before progressing into middle and older ages [16,17]. Therefore, the early detection of carotid intima-media thickness by ultrasonography and establishing the main risk factors in apparently healthy people is imperative for research on atherosclerotic diseases and preventive measures [18,19]. 

Epidemiological, clinical, and morphological studies have shown that the atherosclerotic process begins in the womb by increasing carotid intima-media thickness in fetuses and infants, which significantly boosts the prevalence and progression of the atherosclerotic process in children and adolescents. The increase in the thickness of the carotid intima represents the initial stage of atherogenesis and the base under which the accumulation of lipids with the formation of atherosclerotic plaque is possible [13].

The first evidence of the early origin of atherosclerosis was in an autopsy study performed on American soldiers killed in action in Korea, in which 300 autopsies were performed. The average age was 22.1 years; the youngest recorded age was 18 and the oldest 48 [20]. Over 70% of them had evidence of atherosclerosis in the coronary arteries. In a study conducted in post-World War I Eastern Europe, extended aortic fatty streaks were present in children [16]. Another study demonstrated a very high incidence of “foam cells” in the intima of arterial walls in adolescents [21]. In a study conducted on American children killed in motor accidents, over 50% of them had evidence of early atherosclerosis [21]. A study in Japan (on individuals aged 1 month to 39 years) discovered the presence of “fatty streaks” in 29% of aortas in infants under 1 year old and in 3.1% of coronary arteries in children from 1 to 9 years old [22]. There is overwhelming evidence that primary atherosclerosis prophylaxis measures should include children and adolescents [16]. Epidemiological studies in the United States of America reported aortic fatty streaks in most children over the age of 3 [23].

## 3. Pathophysiology

Atherosclerosis is a chronic, progressive, immuno-inflammatory, and fibro-proliferative disease characterized by changes in the intima of the medium-sized and large arteries caused by lipids aggregation, complex carbohydrates, fibrous tissue, lipid blood components, calcium salts depositions, and the underlying changes of the arterial wall. 

Cholesterol, triglycerides, phospholipids, and free fatty acids are the main lipid blood components [13]. LDL cholesterol and VLDL cholesterol are the most atherogenic lipoproteins. These lipoproteins induce atherogenesis by changing the properties of the endothelium, promoting cell adhesion, and inducing the production of monocytes, macrophages, and the proliferation of smooth muscle cells [24]. Fenton reactions (metal ion catalysis) in the intima lead to reactive oxygen species that can modify the structure of LDL particles, transforming them into oxidized LDL particles [25]. These modified particles of LDL play a pivotal role in the initiation of atherogenesis, facilitating foam cell formation [26,27]. HDL cholesterol can decrease the risk of atherosclerosis and prevent and protect against it [28]. Lipoprotein (a) represents a conjugate particle of LDL cholesterol with apoprotein A and it can suppress the process of fibrinolysis and develop pro-inflammatory effects that will maintain atherogenesis [29,30]. 

Both innate and adaptive inflammatory responses trigger and, in turn, are maintained by atherosclerosis [8,31,32,33]. Monocytes play central roles in the process of atherogenesis by differentiating into macrophages [34]. Lipid accumulation in macrophages induces inflammation, and inflammation stimulates and increases atherosclerotic progression by the formation of typical “foam cells” [35], which can lead to plaque instability under the influence of MMP-9 (matrix metalloproteinase 9) [36]. 

Two subsets of monocytes are associated with the process of atherosclerosis. The “classical monocytes”—CD14^high^ and CD16—which express high levels of chemokine receptor CCR2 and low levels of CX3CR1 lead to chronic inflammation [37,38]. The “non-classical monocytes”—CD14^dim^ CD16+—which express low levels of chemokine receptor CCR2 can contribute to scarring [39]. Therefore, the “classical” monocytes promote atherosclerosis and are associated with cardiovascular events [40,41]. The adaptive immunity is exerted by dendritic cells and T lymphocytes, which promote atherogenesis. The increased histocompatibility complex class II—(HLA-DR) expression on T-cells isolated from unstable plaques proves that within the atherosclerotic lesions, T-cells exhibit an activated profile, promoting further inflammation and local damage [42]. The majority of T cells found in human atherosclerotic plaques are CD4+ T-helper (Th) [42,43] and CD8+ T-helper [42,44,45,46]. Then, CD4+ Th differentiates into Th1, Th2, and the newest lineage, Th17 [47]. Only Th1 was increased in the plasma of patients with coronary artery disease [48]. These promote atherosclerosis by the production of α-interferon, IL-12, and IL-18 [49,50,51]. Th17 cells have an important proatherogenic role and seem to be activated as a response to auto-antigens such as oxidized LDL [52,53]. Th2 cells were shown to enhance inflammation [54,55]. Regulatory T cells (Treg) seem to mitigate atherogenesis [56]. 

In the process of atherosclerosis, activated chemokines and their receptors, such as CCL2-CCR2, CX3C-CX3CL1-CX3C-CX3CR1, CCL5-CCR5, CCL19/CCL21-CCR7, are associated with a high risk of cardiovascular disease [57,58,59,60].

Resident cells are also implicated in the process of atherosclerosis. 

The endothelium constitutes a selectively permeable barrier between blood and tissues. It has sensory and executive functions that can regulate vascular tone and remodeling, but also inflammation and thrombosis [61]. Depending on fluid shear stress, the endothelial cells can have two distinct morphologies: endothelial cells in a region with tubular anatomy, where the blood flow is laminar, have an ellipsoid shape, while in a region with curvature anatomy, where the blood flow is turbulent, endothelial cells have a polygonal shape [62]. Endothelial dysfunction induced by pro-inflammatory stimuli such as accumulation of oxidized LDL, hypercholesterolemia, tobacco use, type 2 diabetes mellitus, or arterial hypertension facilitates atherosclerosis initiation [63,64]. Hypercholesterolemia constitutes a critical trigger to endothelial dysfunction, which leads to impaired nitric oxide response and a reduction in the bioavailability of nitric oxide synthase cofactors [65]. Endothelial dysfunction also leads to the deficiency of some endothelial cell factors, such as angiopoietin-like protein 2 [66], Kruppel-like factor 2 and 4 [67,68], type 1 insulin-like growth factor-1 receptor [69,70], bone morphogenic protein 4 and its receptors [71], which demonstrates that endothelial cells contribute to atherosclerotic lesion formation [72]. 

Vascular smooth muscle cells represent the most predominant type of cells present in the media of the arterial wall [73]. Under the control of platelet-derived growth factor, angiotensin II, and other factors, these cells migrate from the media into the intima of the arterial wall and play a crucial role in the generation of collagen. This migration can contribute to the accumulation of vascular smooth muscle cells in the atherosclerotic plaque. These cells can proliferate for decades. The elaborate extracellular matrix, containing interstitial collagen, elastin, proteoglycans, and glycosaminoglycans, aggravates chronic inflammation [74].

The early lesion of atherosclerosis consists of the accumulation of lipids or lipid-engorged macrophages, called “foam cells” [75]. Recent studies suggest that the precursors of lipid-laden foam cells are represented by macrophages and the metaplasia of the vascular smooth muscle cells [76]. The initial lesion “fatty streaks” can usually be found in the aorta in the first decade of life and in the coronary arteries in the second decade. These lesions appear in vulnerable sites and have no initial clinical expression, causing intense debate about the significance of the fatty streaks [77]. The aortic fatty streaks can usually be found in the thoracic and abdominal aortic segments. The most common sites for the coronary fatty streaks are the medial wall of the left coronary artery, the bifurcation of the circumflex artery, and the medial and posterior sides of the proximal fourth of the anterior descending artery [78].

Although cited in the literature, intermediate lesions between fatty streaks and fibrous plaques are difficult to identify by gross examination [79,80,81]. The highest risk for advanced atherosclerosis is in the intima of the coronary arteries of White male children, adolescents, and young adults, which have more cellular infiltration and connective tissue than the intima of the coronary arteries of women, irrespective of race, or Black patients [82,83].

Following foam cell formation, there is a migration of vascular smooth muscle cells from the media to the intima of the arteries which leads to the formation of the extracellular matrix and which, accompanied by a growing mass of cholesterol and its ester, leads to fibrous plaques [84]. Several risk factors lead to the development of fibrous lesions, such as elevated homocysteine levels [85], arterial hypertension [86], hormones (estrogen has anti-atherogenic functions) [50], and infections, especially cytomegalovirus [87,88]. 

The advanced lesion appears in the fifth to sixth decades of life and depends on the composition and vulnerability of the plaque [89,90]. Vulnerable plaques are associated with a thin fibrous cap with a low density of vascular smooth muscle cells, an increased number of lipids, and macrophages. This thin cap is enveloped by a “lipid-rich” necrotic core. This lesion is known as a vulnerable plaque because of the risk of rupture and thrombosis [71,91]. Rupture frequently occurs at the lesion edges, such as in the proximal anterior descending coronary artery and in the left and right circumflex coronary arteries [6]. Plaque erosion is typically characterized by no endothelium, minimal accumulation of macrophages and T lymphocytes, and an exposed intima containing vascular smooth muscle cells and proteoglycans [6]. 

Clinical trials and genome-wide association studies have all been combined to show that atherosclerosis can be promoted or suppressed by both innate and adaptive immune responses. The progression and remission of atherosclerosis have been linked to a number of inflammatory signaling pathways, including the NLRP3 inflammasome, Notch signaling pathways, toll-like receptors, and proprotein convertase subtilisin/kexin type 9. These pathways are crucial for comprehending the underlying mechanisms of atherosclerosis because they play key roles in the development and reversal of atherosclerosis [92].

## 4. Risk Factors for Atherosclerosis in Children 

Atherosclerosis can develop latently for decades before manifesting clinically in the form of ischemic stroke, peripheral arterial disease, myocardial infarction, and sudden death [93,94]. Epidemiologic studies proved that certain individual characteristics predict the probability that an individual will develop clinical manifestations of atherosclerosis. The risk factors can be divided into modifiable and non-modifiable factors [91], with a possible cumulative effect [93]. Non-modifiable risk factors are represented by age, gender, and family history.

Amongst the main atherosclerosis risk factors in children and adolescents are familial history of hypercholesterolemia, premature cardiovascular diseases, arterial hypertension, and type 1 and 2 diabetes mellitus, as well as personal history of chronic kidney disease, hypothyroidism, systemic lupus erythematous, congenital heart disease, HIV infection, radically cured neoplasms, and organ transplantation [94,95,96,97]. It is recommended to screen children and adolescents who have at least one of the following risk factors: (1) family history of early CVD in a male first-degree relative before the age of 55 or a female first-degree relative before the age of 65, (2) high cholesterol and high lipids, (3) diabetes mellitus or insulin resistance, (4) being overweight/obese, and (5) arterial hypertension.

In 1985, a group of investigators conducted the pathobiological determinants of atherosclerosis in youth (PDAY) study in order to obtain more information about the relationship between risk factors in youth and early clinical manifestation of atherosclerosis [98]. About 300 participants between 15 and 34 years old who died of accidental injuries were investigated by gross examination, histochemical and chemical analyses, and laboratory examination, measuring total and HDL cholesterol postmortem, thiocyanate, glycosylated hemoglobin, and others [99,100]. The initial lesion was observed between 15–19 years of age, when fatty streaks caused luminal narrowing of the thoracic and abdominal aorta by 25% and the right coronary artery by 2% [98]. The PDAY study that evaluated young people helped elaborate a risk score that allows for calculating the effects based on risk factors of atherosclerosis (Table 1) [23,101,102,103].

Other reports noted that in the abdominal aorta, fatty streaks were more extensive in females, with no gender difference in fibrous plaques. In the coronary arteries, there was no gender difference in fatty streaks, but females had less extensive fibrous plaques [23]. A family history of cardiovascular diseases such as myocardial infarction or stroke at an early age represents an individual risk factor for coronary events, especially in adolescents and young adults [104]. 

Having a family history of early heart disease increases the likelihood of atherosclerosis development. Age plays a role as the cumulative effects of risk factors over time can lead to atherosclerosis progression. Males tend to have a higher risk compared to females, although the risk in females increases after menopause. While these factors cannot be modified, awareness of their presence can help identify individuals who may be at higher risk, prompting proactive monitoring and management of modifiable risk factors to reduce the overall risk of atherosclerosis [105].

### 4.1. Arterial Hypertension

Children and adolescents with arterial hypertension are at risk of developing hypertension as adults. Arterial hypertension in children is defined by values ≥ 95th percentile for age for children younger than 13 years and ≥130/80 in children over 13 years old and is associated in the abdominal aorta and right coronary artery with more extensive fibrous plaques [105]. The study conducted by Altay et al. aimed to examine the impact of hypertension on early atherosclerotic alterations and target organ damage in pediatric populations [106]. Hypertension places excessive pressure on the arterial walls, causing damage and promoting the formation of fatty deposits. These deposits can lead to the narrowing and hardening of the arteries, restricting blood flow and potentially resulting in cardiovascular complications. The presence of hypertension during adolescence is concerning as it sets the stage for long-term cardiovascular health risks. Early detection, lifestyle modifications, and appropriate management of hypertension are crucial in reducing the risk of early atherosclerosis and promoting better cardiovascular outcomes in affected adolescents [106].

**Table 1:** Risk factors for atherosclerosis. Scores associated with each factor and probability of occurrence of coronary and abdominal aortic lesions [98,107].

Risk Factors	Coronary Arteries	Abdominal Aorta
**Age (years)**		
15–19 years old	0	0
20–24 years old	5	5
25–29 years old	10	10
30–34 years old	15	15
**Sex**		
Female	−1	1
Male	0	0
**Non-HDL-cholesterol**		
>130 mg/dL	0	0
130–159 mg/dL	2	1
160–189 mg/dL	4	2
190–219 mg/dL	6	3
≥220 μγ/δΛ	8	4
**HDL-cholesterol**		
<40 mg/dL	1	0
40–59 mg/dL	0	0
≥60 μγ/δΛ	−1	0
**Smoking**		
Non-smoker	0	0
Smoker	1	4
**Arterial hypertension**	4	3
**Overweight/Obesity**		
Male		
BMI < 30 kg/m^2^	0	0
BMI > 30 kg/m^2^	6	0
Women		
BMI < 30 kg/m^2^	0	0
BMI > 30 kg/m^2^	0	0
**Diabetes mellitus**		
Glycated hemoglobin (A1c) < 8%	0	0
Glycated hemoglobin (A1c) > 8%	5	3
**Total score**	**Probability of CA lesions**	**Probability of AA lesions**
**0**	0	0
**5**	5	0
**10**	5	2
**15**	10	8
**20**	20	20
**25**	40	50
**30**	60	80

The risk score is calculated by totaling the points for each risk factor. The final score is associated with the probability of having target coronary or abdominal atherosclerotic lesions. The estimated probability of lesions for a given risk score has been detailed in McMahan C et al., 2005. Each score is associated with a probability expressed as a percentage. For example, a risk score of 30, obtained by summing the score for each factor detailed in the previous table, is associated with a 60% probability of coronary lesions and an approximately 80% probability of abdominal aorta lesions [107].

### 4.2. Chronic Inflammation and Immune System Dysregulation

Several studies observed an association between immune activation and inflammation with the risk of early atherosclerosis and eventually stroke and myocardial infarction.

Infectious diseases caused by *Chlamydia pneumoniae*, *Streptococcus pneumoniae*, *Porphyromonas gingivalis*, *Aggregatibacter actinomycetemcomitans*, Human Immunodeficiency Virus, and cytomegalovirus, many of them with increased microbial resistance, have been cited as causes for persistent inflammatory and immune responses [108,109,110].

Obesity, diabetes, or autoimmune disorders are other culprits associated with heightened immune activation and systemic inflammation, further exacerbating the atherosclerosis risk. Both obesity and diabetes can be linked to unhealthy lifestyle factors, including poor diet and sedentary behavior, and contribute to immune dysfunction and promote inflammation. Obesity was associated with more extensive fatty streaks in men in the abdominal aorta and with two-fold more extended fatty streaks in the right coronary arteries between men aged 15 to 24 years, doubling the extension between men aged 25 to 34 years. There was a smaller impact on fatty streaks in the abdominal aorta of the men [111].

Excess body weight, especially abdominal obesity, is associated with several adverse metabolic changes, including dyslipidemia, insulin resistance, and chronic inflammation, which contribute to the development and progression of atherosclerosis. The accumulation of fatty deposits in the arterial walls narrows the blood vessels, impairs blood flow, and increases the risk of cardiovascular events [112]. Addressing these risk factors through healthy lifestyle interventions, such as regular exercise, balanced nutrition, and weight management, is crucial in reducing immune activation, mitigating inflammation, and lowering the risk of early atherosclerosis in adolescents [27].

Immune dysregulation and chronic inflammatory responses are the underlying mechanisms through which depressive syndromes increase the risk for atherosclerosis. High interleukin levels ((IL)-1β, IL-2, IL-6, interferon (IFN)-γ, tumor necrosis factor (TNF)-α, the soluble IL-6 receptor (IL-6R), and the IL-1 receptor antagonist (IL-1RA)) and dysregulation of the hypothalamic-pituitary-adrenocortical (HPA) have been identified in patients with depressive disorders. The former is linked to pro-inflammatory responses and the latter causes high levels of stress hormones [113].

Young women affected by polycystic ovary syndrome may be at increased risk for coronary heart disease, as it is associated with obesity, hyperandrogenism, insulin resistance, and an adverse lipid profile [114].

### 4.3. Dyslipidemia

Dyslipidemia is a primary (congenital) or secondary (acquired) disorder manifested through the elevation of plasma cholesterol and triglycerides. The first person to discover the association between lipid metabolism and cardiovascular events was D.S. Fredrickson in 1950. In 1967, he established the dyslipidemia classification, which has the role of establishing a dyslipidemia type [115]. Therefore, dyslipidemia is split into five types, of which type IIb hyper-lipoproteinemia-hypertriglyceridemia and type IV hyper-pre-lipoproteinemia are more common [115]. Table 2 summarizes normal and pathological lipid levels in children and adolescents.

Type IIb hyper-lipoproteinemia-hypertriglyceridemia occurs in 40% of the cases, and in adults, it is usually associated with metabolic syndrome, type II diabetes mellitus, and combined familial hyperlipidemia. It also occurs in the pediatric population, causing xanthomas, heart diseases, and vegetative-vascular dystonia by the age of 5 in the affected individuals. Type II b dyslipidemia is characterized by an increment of cholesterol and triglyceride levels in blood circulation [115].

Type IV hyper-pre-lipoproteinemia occurs in 45% of cases, affecting both adults and children. Common findings are hepatic steatosis, diabetes mellitus, and lipemia retinalis. Type IV hyper-pre-dyslipidemia is characterized by an increment of VLDL cholesterol and triglyceride levels in blood circulation [13]. The most common hereditary dyslipidemia is represented by familial hypercholesterolemia (FHCH) [116].

When the LDLR (low-density lipoprotein receptors) and PCSK9 (protein-converting subtilisin hexin protein kinase type 9) genes are involved, familial hypercholesterolemia is transmitted in a semi-dominant way, meaning that the proband has a more severe phenotype than both his/her parents. Mutations in the LDLRAP1 (low-density lipoprotein receptor) gene, on the other hand, have a recessive transmission, meaning that the parents of the affected individual have a normal phenotype [13,115,116].

This type of dyslipidemia is characterized by elevated blood levels of LDL cholesterol, which, according to the classification by F. Frederikson, corresponds to type II hyper-lipoproteinemia [13,117].

Heterozygous FHCH occurs with a frequency of 1:200–1:500 and causes LDL cholesterol levels up to 250–500 mg/dL and homozygous FHCH occurs with a frequency of 1:160,000–1:320,000 [118], causing LDL cholesterol levels of 600–1200 mg/dL. The homozygous form is associated with a higher risk of atherosclerotic heart disease than can occur early in life [116,119,120,121]. Heterozygous forms have no clinical markers in children and are usually detected through a routine examination. Clinical manifestations occur at the age of 17 in boys and after the age of 15 in girls [119].

Homozygous forms clinically manifest early, in the first decade of life. The earliest signs are xanthomas, followed by xanthelasmas and atherosclerotic plaques [116,118,120]. Due to chronically increased LDL cholesterol levels after the neonatal period, atherosclerotic changes in the coronary, carotid, and renal arteries occur that can cause aortic insufficiency, aortic, coronary, and renal stenosis, and systemic arterial hypertension [118,121,122]. Due to all of those factors, the homozygous form of familial hypercholesterolemia has a high frequency of sudden cardiac death by acute myocardial infarction in the first or second decade of life [118,123,124,125,126,127,128,129]. Diagnostic scores that use clinical manifestations are helpful in identifying familial hypercholesterolemia [76]. Although the Dutch Lipid Clinic Network score may be a useful tool for physicians diagnosing FHCH, it is limited by the complexity of retrieving all the essential information; a crucial role belongs to clinical judgment in the identification of these subjects [130]. In addition, as demonstrated by Miserez et al. in 2018, phenotypic differences (low-density lipoprotein-receptor gene versus apolipoprotein B-100 gene) might affect the diagnostic value of the Dutch Lipid Clinic Network score and the Simon Broome Diagnostic Criteria [131]. Therefore, in countries with high percentages of FHCH due to apolipoprotein B-100 gene variants, cascade screening and molecular testing are more cost-effective [131]. The diagnostics of atherosclerotic cardiovascular diseases in children, teenagers, and young adults was limited to specialized centers. Due to more invasive and non-invasive advanced techniques, at present it is possible to detect the early formation of atherosclerotic plaques, and visualize the anatomical and mechanical abnormalities of the arteries through coronary angiography, optical coherence tomography, high-resolution B-mode ultrasonography, intravascular ultrasonography, Magnetic Resonance Imaging, or Scintigraphic techniques [132].

**Figure 1:**
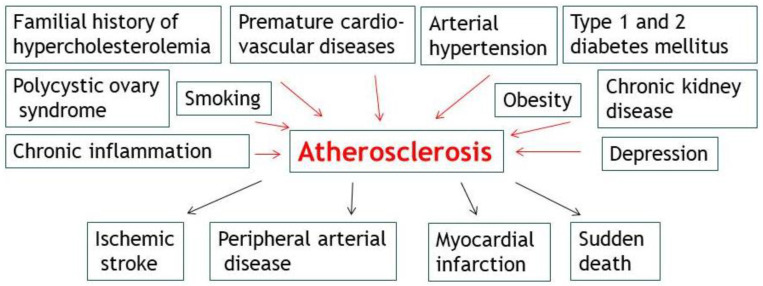
The main risk factors for atherosclerosis and their consequences.

All the previously mentioned risk factors are all the more important as they also represent stress factors in teenagers. Stress and its role in the occurrence of depression must be taken into account when discussing atherosclerosis at young ages. In particular, depression is a leading cause of atherosclerosis, especially in young adult patients [133]. In Figure 1, we summarize the main risk factors for atherosclerosis and their consequences.

### 4.4. Smoking

Smoking and passive exposure to tobacco smoke in association with arterial hypertension, diabetes mellitus, and hyperlipidemia increase the risk of cardiovascular disease and other pathologies [134,135]. Adolescents who smoke are likely to continue the habit into adulthood, with the risk of developing early atherosclerosis, cancer, or other diseases [135,136,137]. Inflammatory processes and oxidative stress have been associated with smoking, and a greater population of macrophage foam cells was isolated in atherosclerotic lesions evaluated in smokers [138,139]. Smoking and pollution expose adolescents to harmful chemicals that damage the lining of blood vessels, leading to inflammation and the formation of fatty deposits. These deposits can restrict blood flow and increase the likelihood of cardiovascular events later in life. The detrimental effects of smoking on arterial health are particularly concerning during adolescence, a critical period for cardiovascular development. Implementing comprehensive tobacco control measures and providing targeted smoking cessation interventions are crucial in reducing the risk of early atherosclerosis among adolescents who smoke [140,141].

In the PDAY study, smoking was associated with extensive abdominal aorta fatty streaks in patients aged 15 to 24 years and three-fold more frequent aortic fibrous plaques in patients aged 25 to 34 years when compared with non-smokers. Smoking also influenced the presence of fatty streaks in the right coronary artery in male patients but to a lesser degree [142].

### 4.5. Systemic Diseases Associated with Increased Atherosclerotic Risk

Certain pediatric disease diseases are associated with considerably accelerated atherosclerosis that involves clinical cardiovascular events in childhood or very early adult life. The American Heart Association has developed three different tiers and each one of them is associated with several different diseases [95,96].

Tier I refers to a high risk of cardiovascular events. This category includes: homozygous familial hypercholesterolemia, type I diabetes mellitus, chronic kidney disease or end-stage renal disease, post-orthotopic heart transplantation, Kawasaki disease, and current coronary aneurysms [50,86]. Nephrotic syndromes are also known causes of atherosclerosis because of the associated dyslipidemia-high LDL, triglycerides, and total cholesterol and low HDL.

Tier II refers to a moderate risk of atherosclerosis that can be manifested by pathophysiological evidence of atherosclerosis. This category includes: heterozygous familial hypercholesterolemia, chronic inflammatory diseases, Kawasaki disease with regressed coronary artery aneurysms, and type II diabetes mellitus [95,96].

Tier III refers to subjects with congenital heart diseases, Kawasaki disease without coronary involvement, and post-treatment cancer survivors [95,96].

## 5. Pharmacologic and Non-Pharmacologic Ways of Dealing with Atherosclerosis

The main modifiable risk factor for atherosclerosis is represented by the dietary intake of cholesterol and saturated and polyunsaturated fat that can be shown by blood cholesterol levels [111,143,144].

Physical activity reduces the risk of atherosclerotic cardiovascular events, including myocardial infarction. There are studies that prove that athletes who perform endurance sports live approximately 3 to 6 years longer than the general population [117,145].

Along with lifestyle changes, pharmacologic treatment should be started as soon as possible. Statins (3-hydroxy-3-methyl-glutaryl-CoA reductase inhibitors), the most common type of substance prescribed, can reduce the LDL cholesterol level in 20–49% of cases, and thereby, the risk of atherosclerotic cardiovascular events is reduced by 22% [146]. Regarding treatment with statins in pediatric patients, there are very few studies, although guidelines recommend starting treatment at the age of 8–10 years, after 6–12 months of diet intervention [147]. Next to lipid-lowering treatments, developing and testing new anti-inflammatory drugs is needed in the future therapeutic approach to cardiovascular disease caused by ATS [148].

Early detection and prevention are essential in managing atherosclerosis in children and reducing the risk of cardiovascular events in adulthood. Screening for traditional risk factors, promoting healthy lifestyle habits, and addressing underlying medical conditions are key strategies in mitigating the progression of atherosclerosis. Furthermore, ongoing research and advancements in the field aim to develop targeted therapies and interventions specifically tailored to pediatric populations, emphasizing the importance of addressing atherosclerosis risk factors in children to promote lifelong cardiovascular health.

## 6. Conclusions

Cardiovascular disease is the leading cause of mortality worldwide, caused mainly by atherosclerosis, which represents the underlying cause of about 50% of all deaths, at least in industrialized countries. During recent years, it has become clear that atherosclerosis is not an inevitable degenerative consequence of ageing, but rather a chronic immune-inflammatory disease of the blood vessels, where different cell types, but mostly endothelial cells, leukocytes, and intimal smooth muscle cells are simultaneously involved in all stages of plaque development. A feared complication of atherosclerosis is plaque rupture, which triggers thrombosis that can lead to several important adverse events, including coronary artery disease, myocardial infraction, stroke, and peripheral artery disease.

Risk factors associated with atherosclerosis, including dyslipidemias, hypertension, diabetes mellitus, obesity, and smoking, are becoming increasingly prevalent in children and adolescents. Inflammation is an independent risk factor for the manifestations of atherosclerosis; however, more research is needed to uncover the genotype-environment interactions at play in this disease. The latest research shows that more and more young people suffer from atherosclerosis, and there is no doubt that an intense prevention shall be started at an early age because atherosclerosis is a primary pediatric problem and should be prevented, managed, and treated efficiently. As a society, we need to endeavor against unhealthy lifestyles and provide a healthy environment to limit the extent of cardiovascular disease in future.

## Figures and Tables

**Table 2:** Assessment of lipid profile in mg/dL and mmol/L in children and adolescents.

Lipids	Levels of Complet Lipid Profile			
	Low	Normal	Borderline	Increased
Cholesterol total	-	<170 mg/dL	170–199 mg/dL	≥200 mg/dL
LDL-cholesterol	-	<110 mg/dL	110–129 md/dL	≥130 mg/dL
Non-HDL-cholesterol	-	<120 mg/dL	120–144 mg/dL	≥145 mg/dL
HDL-cholesterol	<35 mg/dL	>45 mg/dL	35–45 mg/dL	-
Apoprotein A1	<115 mg/dL	>120 mg/dL	115–120 mg/dL	-
Apoprotein B	-	<90 mg/dL	90–109 mg/dL	≥110 mg/dL
Lipoprotein (a)	-	<30 mg/dL	-	≥30 mg/dL
Triglycerides	-	-	-	-
From 0 to 9 years old	-	<75 mg/dL	75–99 mg/dL	≥100 mg/dL
From 9 to 19 years old	-	<90 mg/dL	90–129 mg/dL	≥130 mg/dL
